# The relationship between self-reported borderline personality features and prospective illness course in bipolar disorder

**DOI:** 10.1186/s40345-017-0100-x

**Published:** 2017-09-25

**Authors:** Georg Riemann, Nadine Weisscher, Robert M. Post, Lori Altshuler, Susan McElroy, Marc A. Frye, Paul E. Keck, Gabriele S. Leverich, Trisha Suppes, Heinz Grunze, Willem A. Nolen, Ralph W. Kupka

**Affiliations:** 1 Saxion, University of Applied Science, Handelskade 75, 7417 DH Deventer, The Netherlands; 2 Dimence Mental Health, Center for Bipolar Disorders, Deventer, The Netherlands; 3 GGZ Centraal, Center for Mental Health, Hilversum, The Netherlands; 4 Bipolar Collaborative Network, 5415 W. Cedar Ln, Suite 201-B, Bethesda, MD 20814 USA; 5 Psychiatry and Behavioral Sciences, George Washington University, Washington, DC USA; 6 Former Head UCLA Mood Disorders Research Program, VA Medical Center, Los Angeles, CA USA; 7 Lindner Center of HOPE, Mason, OH USA; 8 Biological Psychiatry Program, University of Cincinnati Medical College, Cincinnati, OH USA; 9 Psychiatry, Mayo Clinic, Rochester, MI USA; 10 Psychiatry & Neuroscience, University of Cincinnati College of Medicine, Cincinnati, OH USA; 11 Department of Psychiatry and Behavioral Sciences, Stanford University School of Medicine, Palo Alto, CA USA; 12 Paracelsus Medical University, Salzburg, Austria; 13 University Medical Center, University of Groningen, Groningen, The Netherlands; 14 Department of Psychiatry, VU University Medical Center, Amsterdam, The Netherlands; 15 GGZ inGeest, Center for Mental Health Care, Amsterdam, The Netherlands; 16 Altrecht Institute for Mental Health Care, Utrecht, The Netherlands

**Keywords:** Bipolar disorder, Borderline personality disorder, Illness course, Life chart methodology

## Abstract

**Background:**

Although bipolar disorder (BD) and borderline personality disorder (BPD) share clinical characteristics and frequently co-occur, their interrelationship is controversial. Especially, the differentiation of rapid cycling BD and BPD can be troublesome. This study investigates the relationship between borderline personality features (BPF) and prospective illness course in patients with BD, and explores the effects of current mood state on self-reported BPF profiles.

**Methods:**

The study included 375 patients who participated in the former Stanley Foundation Bipolar Network. All patients met DSM-IV criteria for bipolar-I disorder (*n* = 294), bipolar-II disorder (*n* = 72) or bipolar disorder NOS (*n* = 9). BPF were assessed with the self-rated Personality Diagnostic Questionnaire. Illness course was based on 1-year clinician rated prospective daily mood ratings with the life chart methodology. Regression analyses were used to estimate the relationships among these variables.

**Results:**

Although correlations were weak, results showed that having more BPF at baseline is associated with a higher episode frequency during subsequent 1-year follow-up. Of the nine BPF, affective instability, impulsivity, and self-mutilation/suicidality showed a relationship to full-duration as well as brief episode frequency. In contrast all other BPF were not related to episode frequency.

**Conclusions:**

Having more BPF was associated with an unfavorable illness course of BD. Affective instability, impulsivity, and self-mutilation/suicidality are associated with both rapid cycling BD and BPD. Still, many core features of BPD show no relationship to rapid cycling BD and can help in the differential diagnosis.

## Background

Bipolar mood disorder (BD) and borderline personality disorder (BPD) are severe psychiatric disorders characterized by a chronic and recurrent illness course. Both disorders have a considerable impact on daily functioning and quality of life and necessitate long-term treatment in most patients. Lifetime-prevalence of BD in an American population based on DSM-IV criteria was 2.4% (Merikangas et al. 2007). Point-prevalence of BPD based on a US-American sample was 1.6% (Lenzenweger et al. 2007). Moreover, there is a considerable co-occurrence of personality disorders (PD) and BD. The prevalence of any PD in patients with BD is estimated between 30 and 40% (Dunayevich et al. 2000; Garno et al. 2005; George et al. 2003; Kay et al. 2002; Schiavone et al. 2004). This concerns mainly cluster B and C personality disorders and in particular BPD. A literature review (Paris et al. 2007) reported a prevalence of bipolar I disorder (BD-I) in patients with BPD ranging from 5.6 to 16.1% (median 9.2%) in eight studies (Pope et al. 1983; McGlashan 1986; Links et al. 1988; Alnæs and Torgersen 1991; Hudziak et al. 1996; Zimmerman and Mattia 1999; Deltito et al. 2001; McGlashan et al. 2000), and a prevalence of bipolar II disorder (BD-II) ranging from 8 to 19% (median 10.7%) in six studies (Links et al. 1988; Zimmerman and Mattia 1999; Deltito et al. 2001; McGlashan et al. 2000; Zanarini et al. 1998; Akiskal 1992). According to these authors, the most methodologically rigorous study (McGlashan et al. 2000) found that 12% of BPD patients met criteria for BD-I, and another 8% met criteria for BD-II. Conversely, they found that 0.5–30% (median 10.7%) of BD-I patients in 12 studies met criteria for BPD (George et al. 2003; Alnæs and Torgersen 1991; Gaviria et al. 1982; Koenigsberg et al. 2002; Jackson et al. 1991; Pica et al. 1990; O’Connell et al. 1991; Turley et al. 1992; Ucok et al. 1998; Vieta et al. 2001; Rossi et al. 2001; Brieger et al. 2003), as well as 12–23% (median 16%) of BD-II patients in three studies (Peselow et al. 1995; Vieta et al. 1999; Benazzi 2000). Paris et al. (2007) concluded that nearly 20% of the patients diagnosed with either BD or BPD also met criteria for the other diagnosis.

According to DSM-IV diagnostic criteria, BPD and BD share phenomenological characteristics with mood instability as the most prominent overlapping feature.

In clinical practice, it can be difficult to differentiate between mood instability that is associated with BPD and the mood fluctuations that occur in rapid cycling BD. Especially, differentiation between BPD and BD-II or unstable forms of BD such as (ultra) rapid cycling can be difficult. Controversy exists whether BPD and BD represent distinct entities or can be seen as part of one psychopathological spectrum (Benazzi 2006; Deltito et al. 2001; Akiskal 2004; Perugi et al. 2003). Still, both disorders need a different therapeutic approach, with more emphasis on psychotherapy in BPD and more on pharmacotherapy in BD.

Co-occurrence of BD and BPD may further complicate the diagnosis and treatment in a given patient. Most studies of PD in patients with BD report that comorbid PD has an unfavorable effect on the course of BD. Moreover, there is evidence that the presence of BPD in patients diagnosed with BD is linked with histories of childhood emotional abuse, physical abuse, and emotional neglect, which may further worsen overall outcome (Garno et al. 2005). Cluster BPD comorbidity was associated with significantly more lifetime suicide attempts and current depression (Garno et al. 2005). A recent literature review concluded that comorbidity of PD in patients with BD is associated with a more complicated course of illness, such as earlier age at onset, longer episodes, and less time euthymic, and increased rates of substance abuse, suicidality, and aggression (Latalova et al. 2013). This was particularly present in BD patients with comorbid BPD (Latalova et al. 2013).

Less is known about the impact of the nine individual DSM-IV borderline personality features (BPF) on illness course of BD, even if patients do not meet full criteria for BPD. A recent study (Fonseka et al. 2015) exploring correlations of borderline personality spectrum symptoms (BPSS) in adolescents with BD showed that high rates of BPSS (identity confusion, interpersonal problems, impulsivity, and emotional lability) was associated with greater mood symptom burden and functional impairment, although in that study no differentiation was made between individual BPF. Another study (Boen et al. 2015) found different profiles on a self-assessment of impulsivity in BPD and BD, whereas BPD patients exhibited markedly elevated scores of impulsivity compared to BD-II patients and healthy controls. In terms of illness course, suicidality is the most studied symptom in BD with comorbid BPD. A study from the Rhode Island Methods to Improve Diagnostic Assessment and Services (MIDAS) concluded that compared to bipolar patients without BPD, patients diagnosed with both BD and BPD were significantly more likely to have made a prior suicide attempt (Zimmerman et al. 2014). Another study (Zeng et al. 2015) found that among patients with severe mood disorders (major depressive disorder, BD or schizoaffective disorder), the presence of comorbid BPF or BPD substantially increased the risk of suicide attempts.

### Aims of the study

To gain further insight in the association between rapid cycling BD and BPD, we investigated the prevalence of the nine BPF in relationship to prospectively assessed mood episode frequency in outpatients with BD.

## Methods

### Sample

The study used data from the Stanley Foundation Bipolar Network (SFBN), a longitudinal naturalistic follow-up study of a large cohort of patients with BD (BD-I, BD-II, and BD-NOS). Data were obtained from patients with BD-I, BD-II, or BD-NOS, who completed at least one full year of daily prospective mood ratings after entering the study. This sample (*n* = 539) was described in detail previously (Kupka et al. 2005). Of this subset those patients who had completed the Personality Disorder Questionnaire (PDQ-4+) as well as mood ratings at baseline were included in the present study (*n* = 375). There were no baseline differences in overall characteristics as shown Table 1 between this sample and the original sample as described in (Kupka et al. 2005).

**Table 1:** Demographic and clinical characteristics

Variable	
Bipolar subtype, *n* (%)	
BD-I	294 (78.4)
BD-II	72 (19.2)
NOS	9 (2.4)
Current mood state, *n* (%)	
Euthymic	169 (45.1)
Manic or hypomanic	16 (4.3)
Depressed	163 (43.5)
Mixed depression and (hypo)manic	27 (7.2)
Demographics	
Ages in years, mean (SD)	42.79 (11.44)
Female gender, *n* (%)	216 (57.6)
GAF score, past week at study entry	64.26 (13.12)
Age first symptoms, years, mean (SD)	19.62 (9.52)
Duration of illness, years, mean (SD)	22.68 (12.32)

*BD* bipolar disorder, *NOS* not otherwise specified, *GAF* global assessment of functioning

### Procedure and instruments

Patients were recruited from private, academic, and community outpatient settings by referral and advertisements. All patients were diagnosed with BD-I, BD-II, or BD-NOS according to DSM-IV criteria. Participants were included if they were 18 years or older, were able to perform daily mood ratings, and were capable of providing written informed consent. Diagnoses of BD and other axis-I diagnoses were made using the Structured Clinical Interview for DSM-IV (First et al. 1995) at baseline.

To assess the presence of BPF, patients completed at baseline the PDQ-4+ (Hyler 1994). The PDQ-4+ assesses all DSM-IV personality disorder criteria by 99 true/false questions. For the current study, we only used the nine DSM-IV BPD features. Episode frequency was calculated by an computer program according to DSM-IV criteria for mania, hypomania, depression, and mixed episodes was based on prospective daily mood ratings with the life chart methodology (LCM) (Kupka et al. 2005; Denicoff et al. 1997). The LCM is a graphic representation of manic and depressive symptom severity and can be used both retrospectively and prospectively. It also provides information about subsyndromal symptoms, medication and psychological treatment, and the presence of possible stressful life events. The LCM was prospectively self-reported on a daily basis and then monthly evaluated and if necessary adjusted by a clinical investigator together with the patient. For this study, both DSM-IV full duration criteria and criteria for brief episodes are used to calculate the number of episodes. According to DSM-IV, following minimum criteria are used to identify mood episodes: 4 days of mild ratings for hypomania, 1 week of moderate ratings or any hospitalization for mania, and 2 weeks of moderate ratings for depression (American Psychiatric Association 2000). In addition, using DSM-IV full duration criterion, an algorithm used in previous NIMH studies as described elsewhere (Kupka et al. 2005; Denicoff et al. 1997) was used to calculate the number of brief duration mood episodes. In short, according to these criterions, a manic episode requires at least 1 day of moderate or severe mania. Depressive episodes were counted if they included at least 2 days of moderate or 1 day of severe depression. In case of switching mood polarity as well as at least 2 weeks of euthymic mood, an episode is considered ended. If euthymic mood lasted less than 2 weeks but was at least 1 day greater than the longest contiguous duration of the adjacent episode, an episode was also considered ended. This method can detect more subtle and short mood switches (Kupka et al. 2005; Denicoff et al. 1997). LCM data of the first prospective year after study baseline were used.

Mood state at baseline and at follow-up was measured by the inventory of depressive symptomatology (Rush et al. 1986; Bernstein et al. 2006) (IDS-SR) and the Young Mania Rating Scale (Young et al. 1978) (YMRS). Depression was defined as an IDS-SR scores of ≥14; (hypo)mania as an YMRS score of ≥12, and mixed states as both IDS-SR ≥14 and YMRS ≥12.

### Statistical methods

Analyses were conducted on all patients who completed all diagnostic assessments at baseline and the subsequent 1-year prospective LCM (*n* = 375). Mood episode frequency was measured continuously. Mood episodes were defined according to both full-duration DSM-IV criteria and brief-duration NIMH-criteria. Regression analyses were used to test the correlation between BPF and episode frequency. Mood state at the moment of rating the PDQ-4+ is tested as a possible confounder on the outcome measure (Kruskal–Wallis test). All statistical analyses were performed by using Statistical Package for the Social Science (SPSS), version 22.

## Results

### Demographic and clinical characteristics

We included 159 (42.4%) males and 216 (57.6%) females with a mean age of 42.8 years (range 19–82), and diagnosed with BD-I (*n* = 294; 78.4%), BD-II (*n* = 72; 19.2%), or BD-NOS (*n* = 9; 2.4%). Self-rated BPD as defined by at least 5 of 9 BPD items on the PDQ-4+ was present in 140 patients (37.3%). Table 1 shows demographic and clinical characteristics.

### Borderline personality features and mood state

**Fig. 1:**
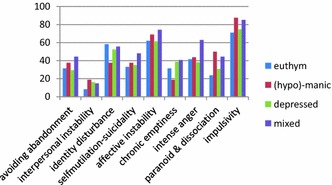
Proportion self-rated BPF profile of patients in various mood states

Current mood state at the time of completing the PDQ-4+ was divided into four groups: euthymic (*n* = 169; 45.1%), hypomanic/manic (*n* = 16; 4.3%), depressed (*n* = 163; 43.5%), and mixed depressed and (hypo)manic (*n* = 27; 7.2%). Figure 1 shows the proportion of self-rated BPF in those mood states. A Kruskal–Wallis one-way ANOVA was performed to examine the effect of mood upon BPF scores. Current mood state had no influence on BPF sumscore measured by PDQ-4+ (*χ* ^2^(3) = 5.533, *p* = .1378). Focusing on individual BPF, we found a significant effect of mood state on the feature paranoid/dissociation. Kruskal–Wallis one-way ANOVA showed a significant group difference (*χ* ^2^(3) = 9.005, *p* = .029). Mean rank was for euthymic (*n* = 160) 177.38, depressed (*n* = 163) 190.52, mixed (*n* = 27) 216.33, and (hypo)manic (*n* = 16) 226.78. No further significant effect on any individual BPF between the groups of euthymic, (hypo)manic, depressed, and mixed patients was found (.596 < *p* > .115).

### Relationship between BPD/BPF and episode

**Fig. 2:**
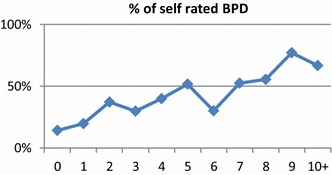
Proportion of bipolar patients with ≥5 self-rated BPD criteria in relationship to prospective full-duration DSM-IV episode frequency

Prevalence of self-reported BPD (≥5 BPF) at baseline increased gradually with increasing episode frequency in the subsequent year (Fig. 2). *T* test showed that there was a significant group difference (*t*(232.63) = −5.80; *p* < 0.01) between patients who had a positive BPD screening (≥5 BPF) and those who had not (<5 BPF). Patients with a positive screening on BPD had more (*M* = 5.64; SD = 4.26) episodes than those who had not (*M* = 3.22; SD = 3.20). Group differences were valid for (hypo-)manic episodes (*t*(226.66) = −4.52; *p* < .01) as well as for depressive episodes (*t*(230.79) = −3.57; *p* < .01). BPD positives had more (hypo-)manic episodes (*M* = 4.44; SD = .356) than BPD negatives (*M* = 2.47; SD = 0.199) and had more depressive episodes (*M* = 4.44; SD = 4.22) than BPD negatives (*M* = 2.47; SD = 3.06). Furthermore, the number of BPF was positively correlated to prospective episode frequency (0 to 10+ episodes/year). Pearson’s product moment revealed that there was significant, although weak, positive correlation between the number of BPF and number of episodes (*r*(375) = .343, *p* < .01). Correlation for (hypo-)manic episodes was stronger (*r*(375) = .301, *p* < .01) than for depressive episodes (*r*(375) = .184, *p* < .01).

### Predictors for unfavorable illness course

**Fig. 3:**
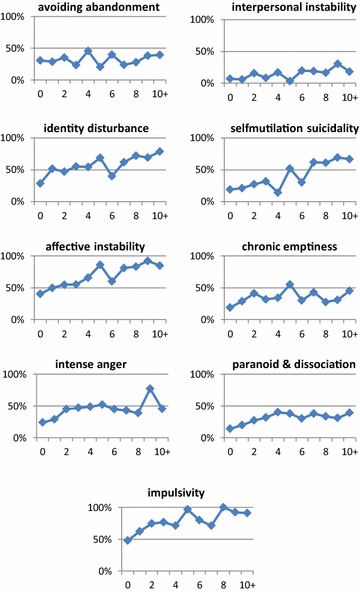
Individual BPF in relationship to full-duration DSM-IV episode frequency. Axis *X* represents the number of episodes; axis *Y* represents proportion of patients with positive BPD screening

A multiple regression of all BPF was conducted to analyses which of the nine BPF at baseline best predicted the total number of full-duration DSM-IV and brief-duration mood episodes at follow-up. Using the stepwise method, we found that affective instability, impulsivity, and self-mutilation/suicidality explain a significant amount of the variance in DSM-IV episode frequencies (*F*(3, 371) = 27.156, *p* < .01, *R* ^2^ = .180, *R*_Adjusted_^2^ = .173). In case of depressive episodes, only self-mutilation/suicidality, chronic emptiness, and interpersonal instability were significant predictors (*F*(3, 371) = 8,82, *p* < .01, *R* ^2^ = .067, *R*_Adjusted_^2^ = .059). In case of hypomanic/manic episodes, only self-mutilation/suicidality and impulsivity were significant predictors (*F*(2, 372) = 13, 72, *p* < .01, *R* ^2^ = .069, *R*_Adjusted_^2^ = .064). Figure 3 shows individual BPF in relationship to the total number of DSM-IV hypomanic, manic, depressive, and mixed episodes. Additionally, a multiple regression analyses of all BPF was conducted to analyses witch BPF can predict brief mood episodes following the NIMH method. We found the same BPF (affective instability, impulsivity, and self-mutilation/suicidality) witch explain the variance in DSM-IV episodes also explain a significant amount of variance in the amount of brief episodes (*F*(3, 371) = 24.200, *p* < .001, *R* ^2^ = .164, *R*_Adjusted_^2^ = .157).

## Discussion

In line with other publications (Latalova et al. 2013; Fonseka et al. 2015), our study confirmed that the presence of BPD is associated with an unfavorable impact on subsequent illness course in BD. In this sample of patients with a primary diagnosis of BD, analyses of correlations showed that there is a positive relationship between the number of BPF at baseline and the number of subsequent mood episodes during 1-year prospective follow-up. Furthermore, analyses of group differences showed that patients who screened positive on BPD at baseline had significant more episodes during the following year than those who screen negative on BPD.

Current mood state when completing the PDQ-4+ had no effect on sumscore of BPF. In our sample, current mood state was not a confounder when analyzing episode frequency. When analyzing the effect of mood state on individual BPF, a single significant difference was found on paranoid/dissociation between euthymic, (hypo)manic, depressed and mixed depressed and (hypo)manic patients. Patients who were (hypo)manic score most on that item, followed by depressed, mixed, and euthymic patients. No further group differences on any other BPF were found.

Focusing on the prediction of an unfavorable illness course especially features related to affective instability had a relevant contribution. We found no differences between predicting full-duration DSM-IV episodes and brief episodes according to the NIMH-algorithm. Of the nine BPF, affective instability, impulsivity, and self-mutilation/suicidality showed a clear relationship to overall mood episode frequency. Our study can not reveal the direction of the relationship between rapid cycling and personality characteristics, i.e., a causal relationship. It may be that a rapid cycling course of BD is driving these personality characteristics or conversely that these personality characteristics induce rapid cycling. In contrast, many core features of BPD such as avoiding abandonment, interpersonal instability, identity disturbance, chronic emptiness, intense anger, and paranoid/dissociation are not related to rapid cycling BD. Our findings suggest that focusing on the shared core phenomenon of mood instability per se, and related phenomena such as impulsivity and suicidality, does not help to differentiate (ultra)rapid cycling BD from BPD. In contrast, one should look for other features of BPD (avoiding abandonment, interpersonal instability, identity disturbance, chronic emptiness, intense anger, and paranoid/dissociation) that are not typically present in rapid cycling BD.

Our study has several limitations. First, and most importantly, the use of a self-reported screening measure of BPD may overestimate the prevalence of BPD. Low agreement has been observed between PDQ-4+ and Structured Clinical Interview for DSM-IV Axis II Disorders (SCID-II) (First et al. 1997), and hence, the PDQ-4+ has been criticized for its tendency to overdiagnose PDs (Fossati et al. 1998). However, this may be more relevant in the detection of full-criteria PD’s than in isolated PD-features. Still, it is plausible that analyses based on BPD ratings obtained from a diagnostic interview for BPD instead of self-report may have yielded different findings. Second, the interpretation of some questions of PDQ-4+ may be somewhat different when answered in the context of BP than BPD. Third, given the naturalistic nature of the study, all patients received state-of-the-art pharmacological treatment tailored to their individual needs. Because of the complexity and high degree of inter-and intra-individual variation among treatment strategies, even during 1 year of follow-up, we could not take this into account in our analyses. The same is true for a highly heterogeneous illness course preceding baseline assessments among participants. Fourth, we focused on episode frequency and did not take into account the severity of illness episodes. Finally, there was no comparison group of patients with a primary or single diagnosis of BPD, although our main outcome measure, mood episode frequency, does not apply to patients with BPD without comorbid mood disorder.

Our study suggests that when differentiating (rapid cycling) BD from BPD, one should rely on those diagnostic features unrelated to mood instability. Our results show that especially avoiding abandonment, interpersonal instability, identity disturbance, chronic emptiness, intense anger, and paranoid/dissociation are features that are not typically present in (rapid cycling) BD. This may be especially relevant in the differentiation of BPD from (rapid cycling) bipolar II disorder, given the difficulty of retrospectively diagnosing hypomania in the absence of a history of mania.

## Abbreviations

BD: bipolar disorder
BPD: borderline personality disorder
BPF: borderline personality features
SFBN: Stanley Foundation Bipolar Network
PDQ-4+: Personality Diagnostic Questionnaire
LCM: life chart methodology
YMRS: Young Mania Rating Scale
IDS-SR: inventory of depressive symptomatology
